# Investigation of rare variation in one thousand cancer genes and its association with risk of breast and prostate cancer

**DOI:** 10.1186/1753-6561-6-S6-P19

**Published:** 2012-10-01

**Authors:** Daniel Klevebring, Johan Lindberg, Simon Sundling, Mårten Neiman, Julia Sandberg, Fredrik Wiklund, Per Hall, Henrik Grönberg, Kamila Czene

**Affiliations:** 1 Department of Medical Epidemiology and Biostatistics, Science for Life Laboratory, Karolinska Institutet, SE-171 77 Stockholm, Sweden; 2 Department of Medical Epidemiology and Biostatistics, Karolinska Institutet, SE-171 77 Stockholm, Sweden

## 

Genome-wide associated studies have provided a first understanding of the genetics behind breast and prostate cancer risk. However, this methodology has limitations in that only previously detected variants can be interrogated, and thus it also is limited to variants with high frequency in a population. Genetic variants detected by genome-wide associated studies each commonly have a low impact on the risk of developing disease. Additionally, a handful of genes have been detected to confer a moderate risk of developing disease.

With the advent of exome sequencing of the genome of various tumor types, it is becoming clear that very few somatically mutated genes (SMGs) are limited to a single tumor type, but rather that most SMGs are found in several tumor types, albeit in different frequencies.

Here we outline a project aimed at investigating germline variation in 1,242 genes known to be somatically mutated in various cancer types, and their association to the risk of breast and prostate cancer.

We have previously developed a methodology to perform a library preparation for sequencing, from genomic DNA to ligated adapters, in a single tube without purification steps (Neiman *et al.* 2012, submitted). Here we modified this protocol to a 96-plate format by ligating a specific barcode in each well in the plate, prior to pooling of wells in batches of 48 samples. This was followed by a sequence capture of the targeted 1,242 genes of interest.

Based on previously collected sample sets where extensive questionnaire data are available for lifestyle factors, family history of cancer et cetera, we selected 480 breast cancer cases and 480 prostate cancer cases with a positive family history and/or young age of onset. Controls for the prostate cancer set were selected to be cancer-free, have a prostate specific antigen of < 1 ng/ml above the age of 56 years. For breast cancer, controls were selected to be cancer-free over the age of 68 years. This extreme design in case/control selection is aimed at enriching for genetic markers of intermediate penetrance for risk of breast and/or prostate cancer in this discovery step of the study.

